# H1 histones: current perspectives and challenges

**DOI:** 10.1093/nar/gkt700

**Published:** 2013-08-14

**Authors:** Sean W. Harshman, Nicolas L. Young, Mark R. Parthun, Michael A. Freitas

**Affiliations:** ^1^Department of Molecular Virology, Immunology and Medical Genetics, The Ohio State University, Columbus, Ohio, USA, ^2^College of Medicine and Arthur G. James Comprehensive Cancer Center, Columbus, Ohio, USA, ^3^National High Magnetic Field Laboratory, Florida State University, Tallahassee, FL, USA and ^4^Molecular and Cellular Biochemistry, The Ohio State University, Columbus, Ohio, USA

## Abstract

H1 and related linker histones are important both for maintenance of higher-order chromatin structure and for the regulation of gene expression. The biology of the linker histones is complex, as they are evolutionarily variable, exist in multiple isoforms and undergo a large variety of posttranslational modifications in their long, unstructured, NH_2_- and COOH-terminal tails. We review recent progress in understanding the structure, genetics and posttranslational modifications of linker histones, with an emphasis on the dynamic interactions of these proteins with DNA and transcriptional regulators. We also discuss various experimental challenges to the study of H1 and related proteins, including limitations of immunological reagents and practical difficulties in the analysis of posttranslational modifications by mass spectrometry.

## CHROMATOSOME STRUCTURE

Histones are evolutionarily conserved proteins responsible for condensation, organization and regulation of the DNA within the nucleus of all eukaryotes. The basic structural element of DNA compaction, the nucleosome core particle, is made up of superhelical DNA wrapped about a protein octamer composed of two copies of each core histone H2A, H2B, H3 and H4 (1–4). Structurally, each core histone has a long central helix with a helix-strand-helix motif on each end forming what is termed the histone fold (5). Hydrophobic interactions between two core histone monomers form heterodimers in a head-to-tail configuration called the handshake motif (2–7). The heterodimers of histones H3 and H4 further associate to form tetramers (5,6). The histone octamer is assembled from two H2A–H2B dimers binding opposite the H3–H4 tetramer (7). Micrococcal nuclease digestion of chromatin exposed to increasing salt concentrations shows symmetrical association of ∼146 base pairs of left-handed superhelical DNA wrapped ∼1.65 turns around the histone octamer forming the nucleosome core particle (5,8–12). Crystallography orients the histone octamer with the H3–H4 tetramer centered between and in direct contact with the DNA entry and exit points and the H2A–H2B tetramer centered opposite. Higher-order chromatin structures are produced through the binding of a linker histone, histone H1, to the nucleosome core particle to form the chromatosome (13–16).

Nucleosomal stabilization facilitated by the chromatosome is provided through the binding of histone H1 to the nucleosomal dyad and the linker DNA entering and exiting the core particle (16–26). Recent •OH radical footprinting experiments show that the positioning of histone H1 at the nucleosomal dyad axis protects an additional 20 base pairs of DNA, 10 base pairs from both the entering and exiting linker DNA, from micrococcal nuclease digestion (8,10,17,25,26). Additional experimental evidence illustrates the influence of histone H1 on chromatin arrangement and compaction (14,19,27–33). However, the specific folding of the 30-nm filament remains controversial and potentially variable in nature (32). In any case, recent studies suggest histone H1 binding provides stabilization and protection through the formation of a dynamic and polymorphic linker histone/linker DNA stem structure (25,26,30,32). Stem-to-stem interactions of neighboring nucleosomes are hypothesized to stabilize folding into higher-order chromatin fibers (26). No matter how the 30-nm chromatin fiber ultimately folds, the influence of histone H1 is dependent on its unique structural characteristics.

## HISTONE H1 STRUCTURE

Histone H1 has a tripartite structure containing an evolutionarily conserved central globular domain with flanking variable domains. X-ray crystallography of the globular domain of the avian erythrocyte linker histone H5 (considered a member of the H1 family) shows a winged-helix motif consisting of three alpha helices with a C-terminal beta hairpin (34). An antiparallel beta sheet is formed between the C-terminal beta hairpin and a short beta strand connecting the first and second alpha helices (34). Conformational studies on the globular domain of the erythrocyte linker histone show that H5 binds asymmetrically to two DNA duplexes through two clusters of highly conserved, positively charged residues on opposite sides of the globular H5 molecule (18,34). Initial positional studies of linker histone H5 on chicken nucleosomes illustrate the globular domain is located between chromatosomal terminal DNA and DNA near the dyad axis of the nucleosome (20). However, more recent experiments using the globular domain of histone H1.5 show binding at the DNA minor groove of the nucleosomal dyad axis (25). As a result, the globular domain has been shown to mediate the protection of 20 additional base pairs of linker DNA by the chromatosome (17,25,26). Although binding of the globular domain of histone H1 can protect almost two full turns of superhelical DNA from micrococcal nuclease digestion, it is the flanking terminal regions of the linker histone that allow for the formation of higher-order chromatin structures (17).

The amino terminus of histone H1 is considered nominally unstructured, as solution and X-ray crystallographic stuctures have yet to be determined (15). Based on sequence, the N-terminus can be divided into two sub-regions (35). The extreme N-terminal sequence is enriched in hydrophobic residues, whereas a highly basic portion resides close to the globular domain (35). The basic cluster has been linked to globular domain positioning and takes on an alpha helical structure in the presence of DNA, whereas the hydrophobic region remains uncharacterized (36,37). Although the N-terminus of bovine thymus histone H1 has been shown to be non-essential for the formation of higher-order chromatin structures, deletion of the N-terminal domain (NTD) of histone H1 isoforms reduces the binding affinity for chromatin *in vitro* (36,38,39). Additionally, histone H1 NTD swapping experiments between mouse H1^o^ and H1c show exchange of their chromatin binding affinities via fluorescence recovery after photobleaching analysis (40). These studies suggest the NTD of histone H1 plays a role in proper binding to the nucleosome. However, additional studies are needed to characterize the functionality of the NTD of histone H1.

Similar to the amino terminus of histone H1, the carboxy terminus lacks X-ray crystallographic resolution and is assumed to nominally be a random-coil, or intrinsically disordered, in solution (41–45). *In vitro* data suggest that on DNA binding at physiological salt concentrations, the C-terminal domain (CTD) of histone H1 takes on a folded conformation dominated by common secondary structural components such as alpha helices, beta sheets, loops and turns (42,45–49). Recent data presented by Fang *et al*. support the formation of secondary structure, as the carboxy terminus of histone H1 settles into DNA helices, allowing for the formation of the nucleosome stem structure (49). Additionally, the interaction of the CTD with linker DNA has been shown to extend beyond the initial 20 base pairs entering and exiting the nucleosome (45).

The CTD accounts for more than half the linker histone sequence, with ∼40% composed of lysine, 20–35% alanine and 15% proline residues (43). Mutational studies on the CTD of histone H1 suggest two distinct functional regions for DNA binding, two 24-amino-acid lengths, facilitate chromatin condensation (44,50). It is hypothesized the remaining CTD length (∼50 amino acids) is involved in protein–protein interactions (44). Support for this concept was recently shown through the binding of DNA methyltransferases (DNMT1 and DNMT3B) to the CTD of mouse histone H1 by Yang *et al*. (51).

The net positive charge imparted on the CTD from the high lysine content allows for regulation of higher-order chromatin structures through DNA backbone charge neutralization (52,53). This allows for low-affinity H1 binding to give rise to the formation of secondary structure in the CTD that permits high-affinity binding (17,36,38,49,50,52,54,55,). In addition to the globular domain, the secondary structure in the CTD enables the formation and stabilization of linker DNA into higher-order chromatin structures (17,25,26,36,38,54,55). The length, charge and number of posttranslational modification (PTM) sites of the C-terminal tails vary between histone H1 isoforms, suggesting that individual H1 variants may play distinct roles in the regulation of higher-order chromatin structure.

## HISTONE H1 GENE FAMILY

The histone H1 gene family in lower organisms is less evolutionarily conserved than that of the core histones. For example, in *Saccharomyces cerevisiae*, the sequence homology between Hho1, the *S. cerevisiae* histone H1 homolog, and *Homo sapien* H1 is 31% identical and 44% similar, whereas histone H4 between the species is 92% identical and 96% similar. Conversely, in higher-order organisms such as the *Gallus gallus* (chicken), the erythrocyte linker histone, H5, shows high sequence homology (66%) to the human histone H1.0, with the greatest sequence divergence found in the CTD (56). In addition to sequence variation, histone H1 proteins also display a range of structures. For instance, *S. cerevisiae* Hho1p contains two globular domains, whereas *Tetrahymena* completely lacks a globular domain (57,58). Eukaryotes also differ in the number of histone H1 variants present. *H. sapiens* and *Mus musculus* both have 11 distinct variants, whereas *Caenorhabditis elegans* has eight and *Xenopus laevis* has five (59). The *H. sapien* family of histone H1 proteins contains five somatic variants (H1.1, H1.2, H1.3, H1.4 and H1.5), which are expressed in nearly all cells (60–62). Six additional H1 variants have been identified in specific tissues, such as H1t and H1T2 in the testis, or cell types, such as H1.0 in terminally differentiated cells (56,63–71). Two types of histone H1 genes exist in human cells: replication-independent and replication-dependent genes. The replication-independent H1 genes, such as histone H1(0), exhibit a replacement phenotype (72). These replacement histone H1s are genomically isolated from other histone genes with transcription based on cellular status (72). Whereas replication-independent H1s have been observed throughout the cell cycle, the majority of the histone H1 protein is produced during S phase of the cell cycle (73–77).

In contrast to replication-independent H1 expression, the replication-dependent variants are found in a large cluster alongside many of the core histone genes located on the short area of chromosome 6 (6p21-p22) (62,78,79). These histone H1 genes, located in gene cluster *HIST1*, have paired expression with DNA replication and core histone mRNA expression levels, although specific H1 variants have been shown to have fluctuating expression across S phase of the cell cycle (77,80–84). The mRNA of replication-dependent H1 genes lack a poly(A) tail and introns commonly observed in other protein-coding genes (85,86). Alternatively, somatic H1 genes contain a 3′ stem-loop sequence allowing for rapid translation during DNA replication, while permitting tight regulation of gene expression after the conclusion of S phase (83,86,87). The expression patterns of individual H1 variants are essential to the functional properties of H1 in the chromatin regulatory system.

In addition to the expression of the normal somatic histone variants, several histone H1 sequence variations have been described. Initially, two sequence variants were described in K562 and Raji cells (88). In the K562 cell line, an alanine to valine substitution is observed at position 17 of histone H1.2 (H1.2A17V) (88). A histone H1.4 sequence variant was found in the Raji cell line corresponding to a lysine to arginine substitution at position 173 (H1.4K173R) (88). Finally, an alanine to threonine substitution at position 142 on histone H1.2 was described by mass spectrometry (MS) in HeLa S3 cells (89). Although identified, the function of the sequence variations remains unknown.

Overexpression of histone H1 variants shows functional differences between the isoforms. Experiments overexpressing histone H1c and H1(0) in the mouse 3T3 cell lines led to distinct phenotypes in these cells. Overexpression of H1(0) results in an increase in nucleosomal repeat length and a decline in cell cycle progression (90,91). Conversely, overexpression of murine histone H1c gives rise to an increase in or no change in transcription levels, while conferring no effect on cell cycle progression (91). These overexpression experiments show functional differences between the two variants, although additional experiments with other isoforms are still needed to further elucidate H1 function.

## HISTONE H1 DYNAMICS

Histone H1 binding to chromatin has been shown to be dynamic in nature, with specific H1 variants divergent in their binding affinity for chromatin (54,55,92–94). It is thought that a high percentage of the total nuclear H1 is bound to nucleosomes at any given time; however, these interactions are individually transient (54,55). Data presented by Lever *et al.* demonstrate *in vivo* dynamics of histone H1.1 occur through soluble intermediates, giving rise to a rapid “stop-and-go” movement of H1.1 in the nucleus between random binding sites (54). Others have further demonstrated that the transient binding of H1 variants with nucleosomes is affected by the structure of the H1 variant, PTMs present on H1 and competition for chromatin binding by other nuclear factors.

Histone H1, as described earlier, has a tripartite structure. Of these, the CTD is the primary determinant of the binding dynamics of each specific variant. For example, fluorescence recovery after photobleaching experiments using NTD green fluorescent protein-tagged H1 variants (H1.0-H1.5) show that the variants with the shortest CTDs have the shortest residence times on nucleosomes (93). Additionally, Th’ng *et al.* show through CTD swapping between H1.1 and H1.4 or H1.5 and truncation experiments with H1.5 that the CTD determines the *in vitro* binding affinity for the nucleosome (93). Similarly, others have shown truncation of histone H1.1’s CTD reduces the residence time of the variant on the nucleosome ∼10-fold *in vivo* (38,54). While CTD length clearly affects variant nucleosomal residence times, the number of phosphorylations and phosphorylation sites also play a role.

Phosphorylation of histone H1 has many distinct functions, leading to both chromatin condensation and decondensation dependent on the site of phosphorylation and cell cycle context. Histone H1 phosphorylation has been shown to progressively increase as a cell progresses from G_1_ to mitosis during the cell cycle (95–101). The overall importance of histone H1 phosphorylation was highlighted by several studies showing that changes in histone H1 phosphorylation can prevent entry into mitosis, thus linking histone H1 phosphorylation with the cell cycle (102,103). The phosphorylation of histone H1 during the cell cycle has been theorized to be a two-fold process (92). First, an interphase (G_0_–S phase) partial phosphorylation that allows for chromatin relaxation and facilitates transcriptional activation (104–107). Second, a maximal phosphorylation during mitosis (M phase) allows for chromatin condensation and separation of chromosomes into daughter cells (95–101). The partial phosphorylation observed in interphase has been shown to induce structural changes in the CTD of H1, which in turn leads to a decreased affinity of histone H1 for DNA (108). Mutational studies mimicking histone H1 phosphorylation have been shown to change the chromatin histone H1 dynamics (109). Additionally, decondensation of chromatin at DNA replication forks has been shown to be a result of histone H1 phosphorylation by cyclin-dependent kinase (CDK) 2 (110). To this end, work by Talasz *et al.* and Sarg *et al.* with histone H1.5 suggests interphase phosphorylation only occurs on serine residues at SPK(A)K sequences (H1.5S17p, H1.5S172p and H1.5S188p) (111,112). Zheng *et al.* have supported this argument by demonstrating H1.2 and H1.4 have serine-only phosphorylation during interphase by MS (H1.2S173p, H1.4S172p and H1.4S187p) (89). The identified interphase phosphorylation sites remained in the mitotic fraction, suggesting preferential hierarchy of phosphorylation on these H1 variants (89). Collectively these studies support the model that interphase phosphorylation on specific histone H1 variants can disrupt DNA–histone interactions, allowing for chromatin relaxation through histone H1 mobilization, and allow for competition and regulation of binding sites on DNA by other nuclear proteins (110,113).

The dynamic nature of histone H1 during interphase allows for regulation of DNA access through several mechanisms (114). First, through condensation of chromatin, histone H1 can limit access of other proteins to chromatin. Lee *et al.* have shown that phosphorylation of histone H1, mimicking H1 removal from chromatin and decondensation, allows for glucocorticoid-induced transcription of the mouse mammary tumor virus promoter (115). Additionally, phosphorylation of histone H1 has been shown to disrupt the interaction between itself and heterochromatin protein 1α, leading to chromatin decondensation (116). Second, histone H1-bound nucleosomes can limit access of chromatin remodeling complexes. For example, the activity of ATP-dependent SWI/SNF chromatin remodeling complexes are reduced and altered when nucleosomes are bound to H1 (117,118). Additionally, this reduction in SWI/SNF activity can be rescued by phosphorylation of histone H1, suggesting a role for histone H1 phosphorylation in chromatin remodeling (119). However, data by Maier *et al.* and Clausell *et al.* have shown that chromatin remodeling complexes can remain active even in the presence of linker histone (24,120). These data suggest specific remodeling complexes can access key nucleosomal elements without the removal of the linker histones. Next, stabilization of the nucleosomal positioning by histone H1 limits the rotational access of specific DNA sequences to transcription factors and other nuclear proteins. This principle was demonstrated by Cheung *et al.*, who showed that estrogen receptor α-mediated transcriptional activity is repressed by H1 via decreased promoter accessibility (121). However, others have demonstrated transcriptional activation of the mouse mammary tumor virus promoter after histone H1 phosphorylation, suggesting a rescue of transcription can be achieved by histone H1 phosphorylation (115,122,123). Finally, histone H1 binding sterically inhibits access of other factors to the chromatin. Herrera *et al.* have demonstrated histone H1 sterically occludes histone acetyl transferase complexes from acetylating the N-terminal tail of histone H3 (124). Whereas interphase phosphorylation of histone H1 is largely involved in transcriptional regulation, mitotic phosphorylation yields a condensed chromatin state allowing for cell division.

The second phase of histone H1 phosphorylation occurs during mitosis. Similar to interphase phosphorylation, mitotic phosphorylation has been shown to be primarily a result of CDK activity at sites of S/TPXK consensus sequences, although non-CDK mitotic phosphorylations have also been identified (Table 1). First described in the 1970s, mitotic phosphorylation of histone H1 is a maximal phosphorylation resulting in the condensation of chromatin (95–101). Several studies by Deterding *et al.* using MS have shown reduction in variant-specific histone H1 phosphorylation in response to therapeutics (CDK inhibitors) and hormones (dexamethasone) (145–147). Furthermore, Th’ng *et al.* have shown through the use of the kinase inhibitor staurosporine that the hyperphosphorylation of histone H1 observed on mitotic chromatin is required to retain condensed chromatin structures (102). Additionally, they established that the inhibition of the H1 kinase by staurosporine arrests cells at the G_2_/M transition, preventing progression into mitosis (102). This study and others, such as those seen with the topoisomerase inhibitor VM-26, emphasize the importance of histone H1 phosphorylation in cell cycle progression (103,148,149). Collectively, these studies suggest the potential for histone H1 kinase inhibitors as cancer therapeutics.

**Table 1.** Histone H1 posttranslational modifications identified by mass spectrometry

H1 variant	Length	Phosphorylation sites	Acetylation sites	Methylation sites	Ubiquitination sites	Formylation sites	References
**H1.2**	213	S2, T4, **T31**, S36, **T146**, **T154**, T165, **S173**	S2^a^, K17, K34, K46, K52, K63, K64, K85, K90, K97, K169, K192	K34, K52, K64, K97, K106, K119, K168, K187	K46, K64, K75, K85, K90, K97, K106	K17, K34, K46, K63, K64, K75, K85, K90, K97, K160	(111,125–137)
H1.3	221	T4, **T18**, S37, **T147**, **T155**, T180, **S189**	S2a, K17, K34, K46, K52, K63, K64, K85, K90, K97, K169	K52, K64, K97, K106, K169	K47, K65, K76, K86, K91, K98, K107	K34, K46, K63, K64, K75, K85, K90, K97, K141, K160	(111,125,126,129,131,132, 135–139)
H1.4	219	S2, T4, **T18**, S27, S36, S41, T142, **T146**, **T154**, **S172**, **S187**	S2a, K17, K26, K34, K46, K52, K63, K64, K85, K90, K97, K169	K26, K52, K64, K97, K106, K119, K148, K169	K17, K21, K34, K46, K64, K75, K85, K90, K97, K106	K17, K34, K46, K63, K64, K75, K85, K90, K97, K110, K140, K160	(111,125,126,128–130,132,133, 135–137,139–142)
H1.5	226	S2, T4, T11, **S18**, T39, S44, S107, **T138**, **T155**, **S173**, **T187**, S189	S2a, K17, K49, K88, K93, K109, K168, K209	K27, K168, K169		K67, K85, K88	(111,125,126,128,129,132, 133,135,137–139,141,143,144)

A list of the posttranslational modifications on the most common histone H1 variants (H1.2, H1.3, H1.4 and H1.5), as identified by mass spectrometry. Phosphorylation sites in bold are consensus CDK sites (S/T-P-X-K, where X is any amino acid).

^a^Denotes *N*-α-acetylation of the N-terminal residue after methionine removal.

An important aspect of histone H1 dynamics that remains unresolved is the degree to which histone chaperones control the dynamics and assembly of the linker histones. Due to their exceptionally high degree of positive charge, the histone proteins can form indiscriminate and deleterious complexes with negatively charged species in the cell such as nucleic acids. A key function of the class of proteins known as histone chaperones is thought to prevent these inappropriate interactions. Although a large number of chaperones have been demonstrated to play a role in core histone transit and assembly, it is not clear whether the movements of histone H1 in the cell and its association with chromatin are mediated by other protein factors or whether they occur spontaneously (150). One potential histone H1 chaperone is the human protein nuclear autoantigenic sperm protein (NASP). NASP has been shown to be associated with both linker and core histones in the cell (151,152). *In vitro*, NASP is capable of binding to histone H1 with nM affinity and to transfer H1 molecules to DNA (153–155). However, a role for NASP in the cellular dynamics of histone H1 has not been directly demonstrated.

## HISTONE H1 POSTTRANSLATIONAL MODIFICATIONS

Although both interphase and mitotic phosphorylation-specific sites have been observed by MS, only a small number of sites have been functionally examined. For example, phosphorylation of H1.4 Ser27 (H1.4S27p) by Aurora B kinase blocks the binding of heterochromatin protein 1α to methylated Lys26 (H1.4K26me), suggesting a cross-talk between these modifications (156,157). Zheng *et al.* showed that interphase phosphorylation at Ser173 on H1.2 (H1.2S173p) and Ser187 on H1.4 (H1.4S187p) is localized to the nucleoli of HeLa S3 cells (89). Phosphorylated Ser187 (H1.4S187p) was further shown to localize to active rDNA promoters, and phosphorylation at this site can be induced by dexamethasone treatment (89). Ser35 phosphorylation on histone H1.4 (H1.4S35p) by protein kinase A mediates H1.4 removal from the mitotic chromatin, suggesting a mechanism of histone H1 mitotic dynamics (158). However, these few examples are not the only sites characterized, and many sites of histone H1 phosphorylations have yet to be functionally described in a site-specific manner.

Although histone H1 phosphorylation is the most researched PTM, other PTMs such as acetylation, methylation and ubiquitination have also been identified (Table 1). The functional relevance of non-phosphorylation PTMs on histone H1 is just coming to light. For example, lysine acetylation at position 34 on histone H1.4 (H1.4K34ac) by the histone acetyltransferase GCN5 has been linked to transcriptional activation and increased dynamic mobility *in vitro* (159). Additionally, further evidence for histone modification cross-talk was shown by the ARTD1-mediated PARylation of histone H3, which induces a shift in specificity of the methyltransferase SET7/9 from H3 to histone H1 (160). Kassner *et al.* further identified new sites of histone H1.4 methylation at Lys-Ala-Lys motifs not previously described (160). The role of other non-phosphorylation PTMs on histone H1 function and dynamic mobility is yet to be explored.

## EXTRACHROMATIN H1 FUNCTION

Beyond the function of histone H1 on chromatin condensation, histone H1 (specifically H1.2) has been found to have an extrachromatin function. Konishi *et al.* found translocation of histone H1.2 to the cytoplasm in response to X-ray-induced DNA double-strand breaks (161). Furthermore, Giné *et al.* show cytosolic movement of H1.2 in chronic lymphocytic leukemia cells after therapeutic intervention (162). The cytosolic histone H1.2 was shown to induce apoptosis through a Bak-mediated mitochondrial release of cytochrome C, allowing for caspase activation (161,162). However, cytosolic histone H1.2 has been observed in non-apoptotic cells as well (14,63) (unpublished data). Collectively, these results suggest there is a mechanism of regulation for histone H1.2 apoptotic induction beyond localization of H1.2 in the cytoplasm. Data presented by Gréen *et al.* and our own unpublished data suggest H1 isoforms are phosphorylated in the cytoplasm of non-apoptotic cells, giving an underlying potential for apoptosis regulation or chaperone binding (163).

## CHALLENGES OF HISTONE H1 ANALYSIS

**Figure 1.**
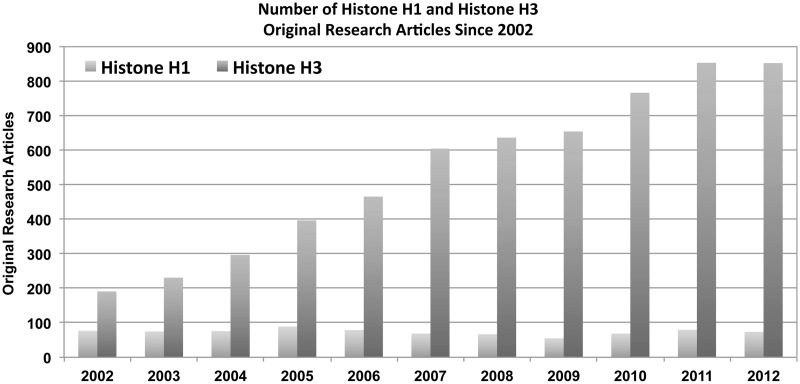
A search of PubMed for primary research articles containing histone H1 or histone H3 in the title or abstract. Data show a steady-state low number (<100) of publications for histone H1, whereas histone H3 displays an increasing trend over time.

Although the work described previously illustrates the successes of research focused on histone H1, progress has been limited for several reasons. Availability and specificity of immunological reagents for histone H1 are drastically lacking. As methods using antibodies are the primary means of molecular and biochemical investigation, limitations in quality antibodies have caused severe difficulty in study. As a result, a lag in understanding of the biological function of histone H1 and its PTMs persists. The production of immunological reagents is hindered by several factors. The demand for histone H1 antibodies remains generally low. A recent search of PubMed for primary research articles with histone H1 in the title or abstract across the past decade revealed a steady-state number (<100/year) of publications (Figure 1). A similar search for the core histone H3 showed an increase in publications over the past decade (Figure 1). These data suggest histone H1 research has not seen the same explosive growth evident for the chromatin field.

We suspect the lull in histone H1 publications can be attributed to two interdependent factors. First, as discussed earlier, histone H1 displays much lower level of evolutionary conservation than the core histones, suggesting that linker histones are not as fundamentally important to chromatin biology. Interest in histone H1 and histone H1 reagents was also dampened by initial studies that suggested that the linker histones were not essential for cell viability. For example, loss of histone H1 genes in *M. musculus*, *T. thermophyla* and *S. cerevisiae* did not affect viability (164–166). However, the essential role of histone H1 in mammals was subsequently affirmed in a landmark publication from the Skoultchi laboratory. Whereas initial single knockout experiments of H1c, H1d and H1e showed no apparent phenotypical changes, a triple knockout of H1c, H1d and H1e (H1.2, H1.3 and H1.4 mouse orthologs) resulted in a 50% reduction in the H1/nucleosome ratio and nucleosome repeat length, ultimately leading to embryonic lethality by E11.5 (167,168). These data suggest a key structural role of histone H1 is to keep the nucleosomes adequately spaced (168,169). Additional *in vitro* work by Fan *et al.* showed triple-H1 knockout in embryonic stem cells leads to substantial changes in chromatin structure with variations in gene expression localized to sites of genes regulated by DNA methylation (27). Findings by Yang *et al.* have confirmed this work by showing histone H1 can interact with DNA-modifying enzymes such as DNMT1 and DNMT3B to alter gene transcription (51). Furthermore, Lee *et al.* have shown a complex of histone H1b (H1.5 mouse ortholog) and the MSX1 transcription factor represses mesoderm differentiation through the regulation of the *MyoD* gene, suggesting a role for specific H1 isoforms in the development of muscle (170).

**Figure 2.**
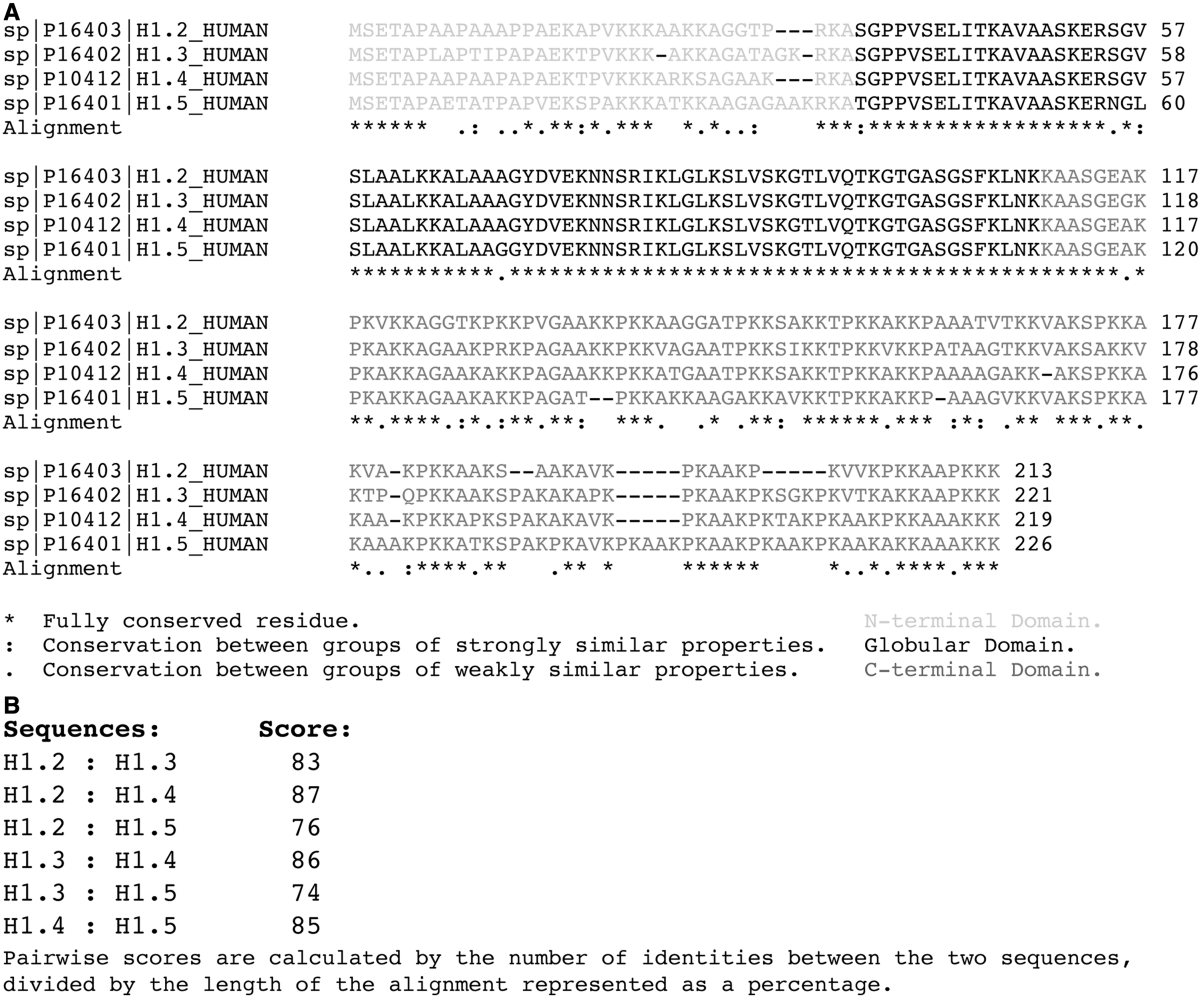
Sequence alignment of histone H1 variants. (**A**) Amino acid sequence alignment for the histone H1 variants H1.2, H1.3, H1.4 and H1.5. (**B**) Pairwise scores of sequence homology. The alignment shows a high homology between the human H1 variants.

Second, the high sequence homology between variants of histone H1 hinders the ability to produce high-specificity antibodies for individual variants. For example, CLUSTAL 2.1 alignment of the four most common somatic histone H1 variant sequences shows high amino acid sequence conservation (Figure 2A) (171). Pairwise scoring of the sequence alignments between variants shows 74–87% sequence homology (Figure 2B). Domain analysis and sequence alignments show the divergence in the sequences of the H1 variants is primarily located at the amino and carboxy termini of the H1 molecule (Figure 2A). As a result, distinction between variants of histone H1 would require partial identification of epitopes from one of these two domains. However, the high number of PTMs on the terminal tails of histone H1 adds additional complexity that could alter the affinity of antibodies for a significant fraction of the molecules in a cell. Despite these complications, there have been a number of recent successes (89,112,143,157,172–174). Importantly, the use of peptides based on the divergent sequences in the NH2-terminal tails of the H1 variants has led to the production of variant-specific antibodies for both chicken and mammalian H1 (173,174). In addition, the generation of phosphorylation-specific H1 antibodies has begun to shed light on the signal transduction pathways that are involved in the modification of the linker histones. For example, Hergeth *et al.* generated a polyclonal antibody against phospho Ser27 of H1.4 (H1.4S27p) and demonstrated this phosphorylation was a result of Aurora B kinase activity (157). Additionally, the Lindner laboratory used the commercially available anti phospho-Thr146 H1 (H1.4T146p) antibody to identify this modification on condensed mitotic chromatin by immunofluorescence (111). Furthermore, Chu *et al.* generated a H1.4 phospho Ser35 (H1.4S35) antibody to show protein kinase A-induced phosphorylation at this site causes removal of H1.4 from the chromatin (158).

**Figure 3.**
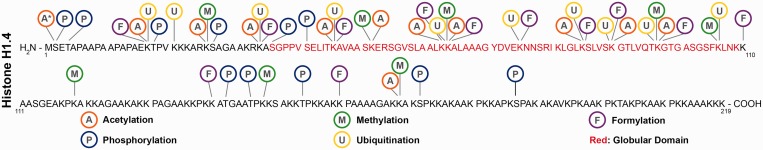
An illustration of the MS-identified posttranslational modifications on histone H1.4. Asterisk denotes *N*-α-acetylation of the N-terminal residue after methionine removal.

The amino and carboxy terminal tails of histone H1 variants are among the most abundantly posttranslationally modified sequences in the cell. For example, Figure 3 depicts the known MS-identified PTMs for the histone H1.4 variant. In line with the data in Figure 3, current literature shows multiple numbers of simultaneous PTMs on histone H1 are regularly identified (125,175). These results suggest antibodies generated toward the tail domains of H1 could result in low specificity based on the PTM combinations present on the H1 molecule and the immunogen used for antibody generation. This effect is similar to that seen with histone H4 and H3 modification-specific antibodies, where antibodies must be generated with distinct combinations of localized PTMs to retain specificity for the epitopes of interest. Consequently, MS has become widely used to analyze histone H1 variants through the ability to bypass the limitations of immunological reagents. However, even MS has limitations when analyzing histone H1.

**Figure 4.**
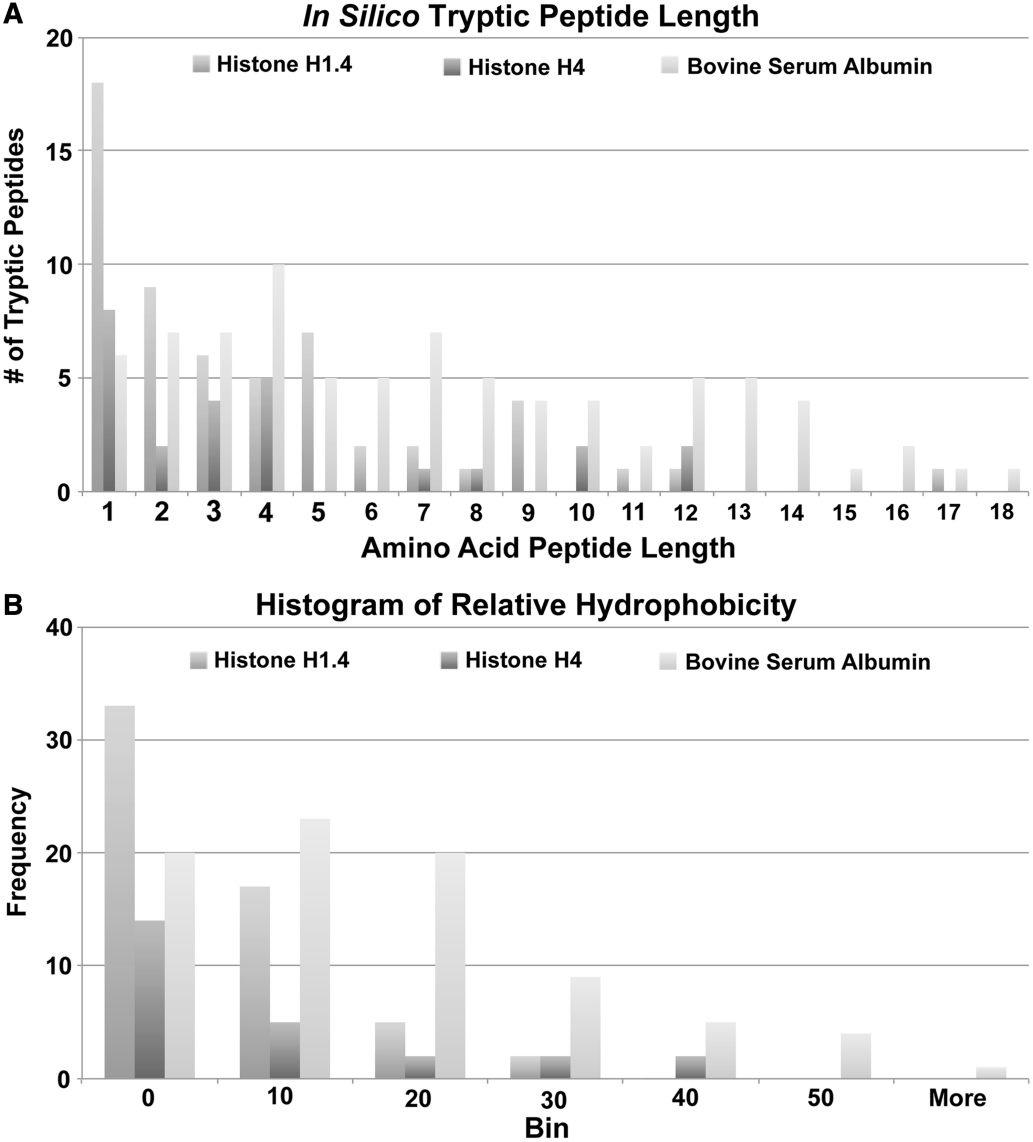
A graphical representation of the tryptic peptide length (**A**) and a histogram of relative hydrophobicities (**B**) for histone H1.4, histone H4 and bovine serum albumin.

Limitations in the use of MS for the analysis of histone H1 result from the inability to use common shotgun proteomic methods for analysis. For example, the most commonly used endoproteinase for shotgun proteomic studies is trypsin. In most commonly expressed soluble proteins, trypsin regularly yields peptides of 6–10 amino acids in length due to the relative abundance of lysine and arginine. Additionally, because trypsin cleaves at the C-terminal side of the basic amino acids, each peptide carries at least two sites for protonation, one at the N-terminal and one at the C-terminal lysine or arginine side chain. Thus, when such a peptide is fragmented via tandem MS, two singly charged ions are typically produced. These properties make trypsin ideal for yielding peptides of a mass and charge suitable for liquid chromatography–electrospray ionization–tandem MS. Although ideal for most soluble proteins, trypsin does not work well for histone H1. Figure 4A is a graphical representation of the peptide lengths generated by an *in silico* digestion of histone H1.4, histone H4 and bovine serum albumin (BSA) with trypsin. The tryptic peptides of H1.4 are very short, with most peptides 5 amino acids or less in length. A similar observation is seen with *in silico* tryptic digest of histone H4. However, BSA yields peptides of variable length more conducive to MS analysis and increased protein sequence coverage (Table 2). Additionally, the peptides that are generated by trypsin for histone H1.4 have low relative hydrophobicities (Figure 4B, Table 2). This results in low retention of peptides on reversed-phase C18 HPLC columns. Furthermore, of those peptides with hydrophobic properties, appropriate masses and peptide lengths generally correspond to the globular domain of the protein. Collectively, these factors lead to poor sequence coverage when compared with more standard proteins such as BSA. As a result, the use of common bottom-up MS strategies with trypsin is limited.

**Table 2.** *In silico* tryptic digestion and relative peptide hydrophobicity

Histone H1.4					Histone H4			Bovine serum albumin		
Position	Domain	Mass (Da)	Tryptic peptide sequence	Relative hydrophobicity	Position	Tryptic peptide sequence	Relative hydrophobicity	Position	Tryptic peptide sequence	Relative hydrophobicity
1–17	NTD	1608.7817	MSETAPAAPAAPAPAEK	17.96	25–36	DNIQGITKPAIR	18.67	45–65	GLVLIAFSQYLQQCPFDEHVK	45.00
35–46	Globular	1197.6605	ASGPPVSELITK	23.86	81–92	TVTAMDVVYALK	34.21	3–19	WVTFISLLLLFSSAYSR	60.49
65–75	Globular	1106.5607	ALAAAGYDVEK	17.53	47–56	ISGLIYEETR	24.65	319–336	DAIPENLPPLTADFAEDK	36.72
111–119	CTD	857.4606	AASGEAKPK	4.9	69–78	DAVTYTEHAK	11.85	169–183	HPYFYAPELLYYANK	33.98
55–63	Globular	844.5018	SGVSLAALK	20.51	61–68	VFLENVIR	31.34	529–544	LFTFHADICTLPDTEK	32.14
98–106	Globular	810.3872	GTGASGSFK	10.76	97–103	TLYGFGG	23.94	508–523	RPCFSALTPDETYVPK	26.62
160–168	CTD	783.4602	KPAAAAGAK	2.81	1–4	MSGR	3.40	469–482	MPCTEDYLSLILNR	42.61
91–97	Globular	745.4334	GTLVQTK	12.49	57–60	GVLK	9.04	184–197	YNGVFQECCQAEDK	19.55
141–148	CTD	715.3864	ATGAATPK	1.04	22–24	VLR		267–280	ECCHGDLLECADDR	18.00
130–136	CTD	641.3860	KPAGAAK	4.22	10–13	GLGK	4.91	347–359	DAFLGSFLYEYSR	44.12
47–52	Globular	545.3173	AVAASK	2.92	42–45	GGVK	3.97	139–151	LKPDPNTLCDEFK	22.23
203–207	CTD	543.3380	TAKPK	2.29	94–96	QGR		438–451	VPQVSTPTLVEVSR	25.49
193–197	CTD	541.3588	AVKPK	1.77	38–40	LAR		387–399	DDPHACYSTVFDK	21.44
86–90	Globular	532.3220	SLVSK	9.08	14–17	GGAK	3.66	421–433	LGEYGFQNALIVR	34.17
178–182	CTD	513.3275	AAKPK	2.27	19–20	HR		569–580	TVMENFVAFVDK	40.25
198–202	CTD	513.3275	AAKPK	2.27	7–9	GGK		375–386	EYEATLEECCAK	19.43
208–212	CTD	513.3275	AAKPK	2.27	5–6	GK		286–297	YICDNQDTISSK	17.06
27–32	NTD	503.2703	SAGAAK	2.47	18	R		106–117	ETYGDMADCCEK	15.45
76–79	Globular	489.2296	NNSR	2.31	37	R		89–100	SLHTLFGDELCK	26.51
18–21	NTD	443.2744	TPVK	4.05	41	R		76–88	TCVADESHAGCEK	6.12
82–85	Globular	429.2951	LGLK	11.37	46	R		402–412	HLVDEPQNLIK	18.84
123–127	CTD	416.2383	AGAAK	2.88	79	R		361–371	HPEYAVSVLLR	25.00
187–190	CTD	401.2274	SPAK	3.28	93	R		300–309	ECCDKPLLEK	13.08
107–109	Globular	373.2325	LNK		21	K		66–75	LVNELTEFAK	28.92
137–139	CTD	371.2532	KPK		80	K		460–468	CCTKPESER	5.07
214–217	CTD	359.2168	AAAK	3.16				588–597	EACFAVEGPK	18.92
154–156	CTD	344.2060	TPK					499–507	CCTESLVNR	13.86
172–174	CTD	330.1903	SPK					310–318	SHCIAEVEK	8.92
184–186	CTD	314.1954	APK					549–557	QTALVELLK	31.48
150–152	CTD	304.1747	SAK					413–420	QNCDQFEK	9.92
53–54	Globular	303.1543	ER					598–607	LVVSTQTALA	22.03
80–81	Globular	259.1896	IK					123–130	NECFLSHK	15.81
24–25	NTD	245.1488	AR					37–44	DLGEEHFK	14.77
120–121	CTD	217.1426	AK					161–167	YLYEIAR	25.57
128–129	CTD	217.1426	AK					249–256	AEFVEVTK	18.96
158–159	CTD	217.1426	AK					131–138	DDSPDLPK	14.11
170–171	CTD	217.1426	AK					483–489	LCVLHEK	14.10
176–177	CTD	217.1426	AK					562–568	ATEEQLK	8.95
191–192	CTD	217.1426	AK					257–263	LVTDLTK	16.78
33	NTD	174.1117	R					341–346	NYQEAK	4.02
22	NTD	146.1055	K					581–587	CCAADDK	3.29
23	NTD	146.1055	K					29–34	SEIAHR	4.83
26	NTD	146.1055	K					212–218	VLASSAR	8.89
34	NTD	146.1055	K					198–204	GACLLPK	17.86
64	Globular	146.1055	K					236–241	AWSVAR	16.55
110	CTD	146.1055	K					490–495	TPVSEK	3.58
122	CTD	146.1055	K					118–122	QEPER	0.97
140	CTD	146.1055	K					205–209	IETMR	11.47
149	CTD	146.1055	K					223–228	CASIQK	6.48
153	CTD	146.1055	K					524–528	AFDEK	8.64
157	CTD	146.1055	K					101–105	VASLR	9.87
169	CTD	146.1055	K					157–160	FWGK	19.96
175	CTD	146.1055	K					281–285	ADLAK	8.03
183	CTD	146.1055	K					558–561	HKPK	1.57
213	CTD	146.1055	K					229–232	FGER	7.76
218	CTD	146.1055	K					25–28	DTHK	2.24
219	CTD	146.1055	K					20–23	GVFR	13.33
								242–245	LSQK	2.96
								337–340	DVCK	3.49
								152–155	ADEK	2.46
								434–436	YTR	
								456–459	VGTR	3.32
								452–455	SLGK	4.66
								246–248	FPK	
								545–547	QIK	
								264–266	VHK	
								496–498	VTK	
								233–235	ALK	
								372–374	LAK	
								219–220	QR	
								35–36	FK	
								221-222	LR	
								1–2	MK	
								210–211	EK	
								298–299	LK	
								400–401	LK	
								24	R	
								168	R	
								360	R	
								156	K	
								437	K	
								548	K	

A list of the sequences, peptide lengths, domain, relative hydrophobicities and masses produced by an *in silico* digestion of histone H1.4 with trypsin. Histone H4 and BSA were also added for comparison.

The use of alternative endoproteinases for middle-down proteomics also yields poor digestion results. For example, endoproteinase Glu-C cleaves proteins at the C-terminal side of glutamic acid in ammonium bicarbonate buffer. *In silico* digests of histone H1.4, histone H4 and BSA with Glu-C, shown in Table 3, yield less-than-optimal peptide lengths and charges. Glu-C digests of histone H1.4 result in long, highly charged peptides not readily suited for liquid chromatography-MS/MS analysis. Additionally, Glu-C cleavage of histone H1.4 gives a peptide encompassing nearly the entire CTD (aa 116–219). Similar results are obtained from *in silico* digests of histone H4. Conversely, Glu-C digests of BSA yield many suitable peptides in length and charge for MS analysis. Collectively, these results suggest the amino acid sequence of histone H1 does not lend to commonly used bottom-up and middle-down MS strategies.

**Table 3.** V8 protease (Glu-C) *in silico* digestion and charge states

Histone H1.4				Histone H4				Bovine serum albumin			
Sequence	Length	Charge (+)	Peptide amino acid composition	Sequence	Length	Charge (+)	Peptide amino acid composition	Sequence	Length	Charge (+)	Peptide amino acid composition
116–219	104	44	AKPKAKKAGAAKAKKPAGAA KKPKKATGAATPKKSAKKTP KKAKKPAAAAGAKKAKSPKK AKAAKPKKAPKSPAKAKAVK PKAAKPKTAKPKAAKPKKAA AKKK	1–54	54	17	MSGRGKGGKGLGKGGAKRHR KVLRDNIQGITKPAIRRLAR RGGVKRISGLIYEE	383–419	37	5	CCAKDDPHACYSTVFDKLKH LVDEPQNLIKQNCDQFE
75–115	41	10	KNNSRIKLGLKSLVSKGTLV QTKGTGASGSFKLNKKAASG E	76–103	28	7	HAKRKTVTAMDVVYALKRQG RTLYGFGG	1–30	30	6	MKWVTFISLLLLFSSAYSRG VFRRDTHKSE
17–42	26	10	KTPVKKKARKSAGAAKRKAS GPPVSE	65–75	11	2	NVIRDAVTYTE	424–448	25	4	YGFQNALIVRYTRKVPQVST PTLVE
54–74	21	4	RSGVSLAALKKALAAAGYDV E	55–64	10	3	TRGVLKVFLE	125–149	25	4	CFLSHKDDSPDLPKLKPDPN TLCDE
4–16	13	1	TAPAAPAAPAPAE					276–300	25	5	CADDRADLAKYICDNQDTIS SKLKE
43–53	11	3	LITKAVAASKE					42–62	21	2	HFKGLVLIAFSQYLQQCPFD E
1–3	3	1	MSE					211–231	21	6	KVLASSARQRLRCASIQKFG E
								232–250	19	6	RALKAWSVARLSQKFPKAE
								528–543	16	2	KLFTFHADICTLPDTE
								449–465	17	3	VSRSLGKVGTRCCTKPE
								503–518	16	3	SLVNRRPCFSALTPDE
								474–488	15	2	DYLSLILNRLCVLHE
								573–588	16	1	NFVAFVDKCCAADDKE
								177–190	14	2	LLYYANKYNGVFQE
								254–267	14	4	VTKLVTDLTKVHKE
								165–176	12	3	IARRHPYFYAPE
								364–375	12	3	YAVSVLLRLAKE
								155–164	10	4	KKFWGKYLYE
								345–356	12	2	AKDAFLGSFLYE
								31–41	11	3	IAHRFKDLGEE
								555–565	11	4	LLKHKPKATEE
								544–554	13	4	KQIKKQTALVE
								595–607	10	2	GPKLVVSTQTALA
								335–344	11	3	DKDVCKNYQE
								324–334	11	1	NLPPLTADFAE
								196–206	10	3	DKGACLLPKIE
								88–97	10	2	KSLHTLFGDE
								107–116	9	1	TYGDMADCCE
								519–527	9	2	TYVPKAFDE
								98–106	9	3	LCKVASLRE
								73–81	7	2	FAKTCVADE
								357–363	8	3	YSRRHPE
								301–308	8	2	CCDKPLLE
								495–502	8	3	KVTKCCTE
								268–275	7	1	CCHGDLLE
								566–572	7	2	QLKTVME
								63–69	7	2	HVKLVNE
								309–315	6	2	KSHCIAE
								468–473	6	2	RMPCTE
								318–323	6	2	KDAIPE
								489–494	6	2	KTPVSE
								589–594	5	1	ACFAVE
								117––121	5	2	KQEPE
								150–154	6	2	FKADE
								82–87	5	1	SHAGCE
								378–382	5	1	ATLEE
								191–195	4	1	CCQAE
								207–210	4	2	TMRE
								420–423	3	2	KLGE
								122–124	3	2	RNE
								251–253	3	1	FVE
								70–72	3	1	LTE
								376–377	2	1	YE
								316–317	2	1	VE
								466–467	2	1	SE

A list of peptide lengths, charge and sequences produced by an *in silico* digestion of histone H1.4, histone H4 and BSA with V8 protease (Glu-C).

The inability to use bottom-up and middle-down approaches has drastically limited the ability to study histone H1 via MS. However, top-down MS techniques, although limited, have been successfully applied to study histone H1 PTMs. For example in *Drosophila melanogaster*, Bonet-Costa *et al.* used top-down MS/MS to map both single and multiple co-existing histone H1 PTMs after collision induced dissociation or electron-capture dissociation (176). Although effectively applied to the single H1 variant in *Drosophila*, top-down MS/MS on human H1 is severely limited by the necessity for high protein purity, high concentration and separation of the multiple variants. As a result, others have used top-down MS to assess the relative abundance of histone H1 PTMs without fragmentation (89,177,178). For instance, Wang *et al.* monitored changes in histone H1.5 phosphorylation patterns after drug treatment in acute myeloid leukemia cell lines using intact mass MS (178). While giving the number of modifications and abundances, these approaches do not yield the specific location of the PTM as top-down MS/MS can. Despite these limitations in proteomic methods for the analysis of histone H1, adaptations of these methods in conjunction with state-of-the-art equipment has led to progress in the study of histone H1.

## FUTURE DIRECTIONS OF FIELD

The immunological limitations for studying the function of histone H1 and its PTMs make it a challenging field of research. Although progress has been made, overcoming these difficulties will require combinatorial mass spectral methods. The use of a top-to-bottom proteomics approach will facilitate targeted characterization of specific histone H1 variants and PTMs of interest where a single MS method may fail. Site-directed mutagenesis and the application of single and multiple reaction monitoring experiments to histone H1 variants will allow for further functional descriptions without the necessity for immunological reagents. Collectively, the use of such methods will unlock the specific cellular functions of each histone H1 variant and their respective PTMs.

## FUNDING

Funding of this review was provided by grants from the National Institutes of Health [R21 DK082634, R01 CA107106 and R01 GM62970]; and support from The Ohio State University. Funding for open access charge: [R01 CA107106 and R01 GM62970].

*Conflict of interest statement*. None declared.
